# Plant growth-promoting rhizobacterial biofertilizers for crop production: The past, present, and future

**DOI:** 10.3389/fpls.2022.1002448

**Published:** 2022-09-16

**Authors:** Becky N. Aloo, Vishal Tripathi, Billy A. Makumba, Ernest R. Mbega

**Affiliations:** ^1^Department of Biological Sciences, University of Eldoret, Eldoret, Kenya; ^2^Department of Biotechnology, GLA University, Mathura, Uttar Pradesh, India; ^3^Department of Biological and Physical Sciences, Moi University, Eldoret, Kenya; ^4^Department of Sustainable Agriculture and Biodiversity Conservation, Nelson Mandela African Institution of Science and Technology, Arusha, Tanzania

**Keywords:** biofertilizers, sustainable agriculture, plant growth-promoting rhizobacteria, microbial stimulants, microbial formulations

## Abstract

Recent decades have witnessed increased agricultural production to match the global demand for food fueled by population increase. Conventional agricultural practices are heavily reliant on artificial fertilizers that have numerous human and environmental health effects. Cognizant of this, sustainability researchers and environmentalists have increased their focus on other crop fertilization mechanisms. Biofertilizers are microbial formulations constituted of indigenous plant growth-promoting rhizobacteria (PGPR) that directly or indirectly promote plant growth through the solubilization of soil nutrients, and the production of plant growth-stimulating hormones and iron-sequestering metabolites called siderophores. Biofertilizers have continually been studied, recommended, and even successfully adopted for the production of many crops in the world. These microbial products hold massive potential as sustainable crop production tools, especially in the wake of climate change that is partly fueled by artificial fertilizers. Despite the growing interest in the technology, its full potential has not yet been achieved and utilization still seems to be in infancy. There is a need to shed light on the past, current, and future prospects of biofertilizers to increase their understanding and utility. This review evaluates the history of PGPR biofertilizers, assesses their present utilization, and critically advocates their future in sustainable crop production. It, therefore, updates our understanding of the evolution of PGPR biofertilizers in crop production. Such information can facilitate the evaluation of their potential and ultimately pave the way for increased exploitation.

## Introduction

The earth will be home to about 10 billion people by 2050 and a lot of pressure will be mounted on the existing food resources (United Nations, 2015). Although global crop production can be achieved through agricultural intensification, this will escalate reliance on chemical agro-inputs like fertilizers that pose several environmental effects (Vassilev et al., 2015; Abhilash et al., 2016b). For instance, chemical fertilizers are extensively associated with greenhouse gas emissions that fuel global warming and climatic changes (e.g., Kahrl et al., 2010; Mapanda et al., 2011; Carmo et al., 2013). Similarly, the eutrophication of several water bodies and the destabilization of aquatic ecosystems have several times been attributed to fertilizer runoffs from agricultural fields (Melo et al., 2012; Deepa and Venkateswaran, 2018; Zhang et al., 2018). Ironically, long-term artificial fertilization can also include the overall deterioration of soil productivity and quality through acidification (Neog, 2018; Bai et al., 2020; Yan et al., 2020).

Owing to the aforementioned challenges, the exploration of alternative crop fertilization mechanisms is mounting worldwide in an attempt to develop sustainable food production systems. The exploitation of plant microbiomes has particularly gathered surmountable interest in this regard. Among the most interesting plant microbiomes are the plant growth-promoting rhizobacteria (PGPR) that present several advantageous functions in plant rhizospheres, fr**o**m nutrients solubilization (Ibarra-Galeana et al., 2017; Borgi et al., 2020; Verma et al., 2020), to suppression of plant diseases (e.g., Rizvi et al., 2017; Agisha et al., 2019; Bektas and Kusek, 2021; Jayakumar et al., 2021), nitrogen (N_2_) fixation (Bahulikar et al., 2014; Hara et al., 2020), and improved phytochemical composition (Rizvi et al., 2022a), among others. Biofertilizers are microbial formulations of PGPR strains that can either be immobilized or trapped on inert carrier materials to enhance plant growth and soil fertility (Aloo et al., 2022a). Over the decades, considerable strides have been made to understand, investigate and formulate various PGPR as alternative crop fertilization tools (e.g., Htwe et al., 2019; Paliya et al., 2019; Bangash et al., 2021; Barin et al., 2022; Aloo et al., 2022b). The yield of various crops can be increased by about 25% and the use of inorganic N and P fertilizers be reduced by about 25–50 and 25% through biofertilizer application (Khan and Chattopadhyay, 2009; Saber et al., 2012).

The utilization of biofertilizers dates back to the 1980s when the first *Rhizobium* formulations were patented and marketed in Germany (Nobbe and Hiltner, 1986). Several developments have been made through the decades and today, biofertilizer formulations are applied for the production of several crops entirely or with reduced usage of artificial fertilizers as presented in Section 4. Despite these developments, biofertilizer technology is yet to be exploited to its maximum potential. It is important to increase our knowledge of biofertilizers and their massive potential in the sustainability of our food production systems to increase their utilization. Herein, we evaluate the history of PGPR biofertilizers, assess their present utilization status from a global perspective, and critically propound on their future in sustainable crop production. This can update our understanding of the evolution of PGPR biofertilizers in crop production. We believe that such information will provide a good starting point for debate, and intensive global efforts to harness these bio-resources as biotechnological-based solutions for sustainable crop production systems. This work has been modified from a previous preprint (Aloo et al., 2020).

## Overview of rhizobacterial biofertilizers and types

The meaning of biofertilizers has evolved for several decades, with many interpretations. The term has therefore received several different definitions over time (Table 1), reflecting the development of our comprehension of them. Most scholars consider PGPR as a biofertilizer because of their positive influences on the plant rhizospheres that can generally stimulate plant growth. However, Riaz et al. (2020) advance that PGPR and biofertilizers should not be used interchangeably since not all PGPR are biofertilizers.

**Table 1:** Common definitions of biofertilizers from different literature.

Literature	Provided definition
Mazid and Khan (2015)	A biologically-active product or microbial inoculant/formulation with one or several beneficial microbes, conserving and mobilizing crop nutrients in soil.
Vessey (2003)	A preparation with one or several microbial species capable of mobilizing essential plant nutrients from non-usable to usable forms.
Malusá et al. (2012)	A formulation with one or several microbes that enhance soil fertility and promote plant growth by availing nutrients and increasing plant access to nutrients.
Bisen et al. (2015)	A unique, environmentally-friendly, and cheap alternative to artificial fertilizers that improve soil health and crop productivity sustainably.
Sahu and Brahmaprakash (2016)	A formulation/preparation with latent/living microorganisms with long-term storage, ease of handling, and delivery of effective microbes from the laboratory to the field for crop application.
Tomer et al. (2016)	A microbial inoculant that colonizes the rhizosphere and improves plant growth by enhancing plant nutrient availability and accessibility.
Simarmata et al. (2016)	A product with several beneficial microbes for improving soil productivity through nitrogen (N) fixation, solubilization of P, and plant growth stimulation through the synthesis of plant growth-promoting (PGP) substances.
Nair and Brahmaprakash (2017)	A mixture/product containing an active ingredient and inactive/inert substances.
Kumar et al. (2014)	A formulation or a biological product that contains microbes that can improve nutrient solubility in soil and fix atmospheric N and/or enhance crop yield.
Bhardwaj et al. (2014)	A formulation made of beneficial microbes and/or biological products and can enhance nutrient solubility in soil or fix atmospheric N and/or has the potential of enhancing crop yield.
Brahmaprakash and Sahu (2012)	A preparation of beneficial microbes that can boost plant growth or fertilizer that can meet the nutritional requirements of crops microbiologically.
Atieno et al. (2020)	Products containing beneficial microorganisms that enhance soil fertility and crop productivity.
Riaz et al. (2020)	Formulations of living microbial cells as single or multiple strains that promote plant growth by increasing nutrient availability and acquisition.

**Figure 1:**
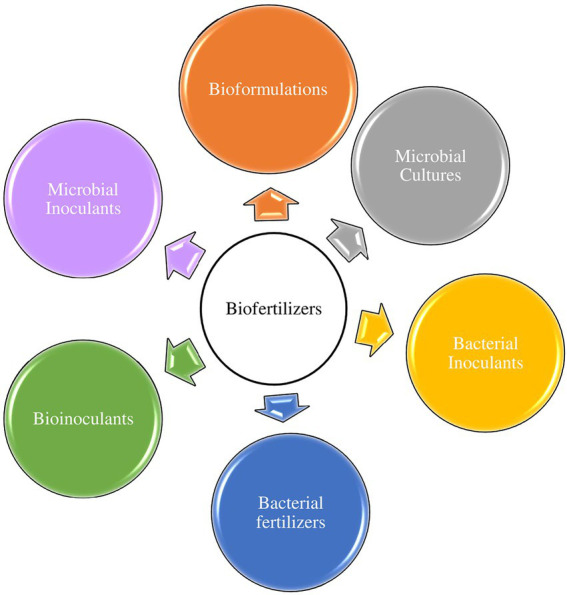
Terminologies used interchangeably with microbial biofertilizers.

This is probably because the efficient PGPR must be formulated into products that can be applied to plants/soils to stimulate plant growth to qualify as biofertilizers. Nevertheless, the major components of biofertilizers are PGPR whose activities generally contribute to the overall increment, concentration, and accessibility of plant nutrients in plant rhizospheres. Herein, we adopt the definition of biofertilizers as active microbial agents that stimulate plant growth by improving nutrient availability in plant rhizosphere(s). Other synonymous terminologies with biofertilizers are microbial inoculants or bioformulations, bioinoculants, microbial cultures, and bacterial fertilizers or inoculants (Figure 1).

**Figure 2:**
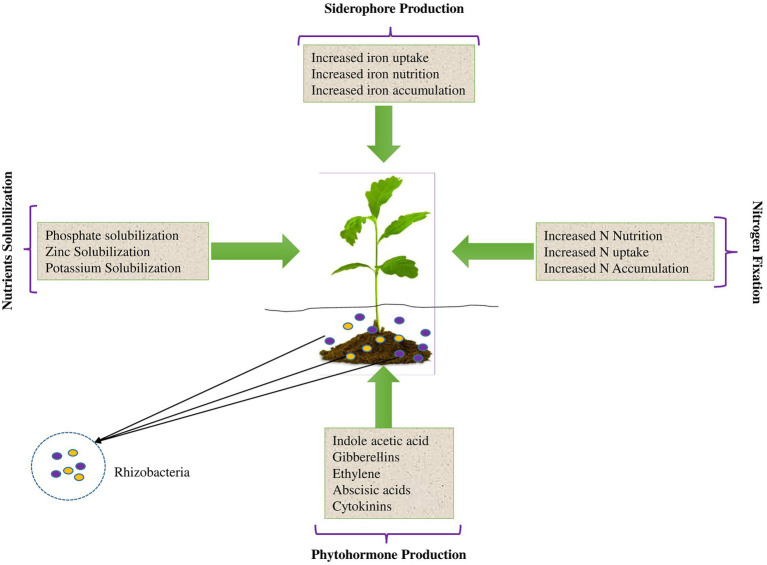
A simulation of the various functions of biofertilizers in plant growth promotion.

There are several types of biofertilizers depending on their functions in plant rhizospheres. Notably, a single biofertilizer can consist of a single PGPR strain with single or multiple PGP traits, or microbial consortia with multifarious PGP traits. A simulation of the various functions of biofertilizers in PGP is shown in Figure 2 and subsections 2.1 to 2.5 highlight the various types of PGPR biofertilizers. Nevertheless, the different types of biofertilizers normally function synergistically and offer an effective and environmentally-friendly solution for achieving food security while minimizing environmental impacts. Consequently, biofertilizers and PGPR are largely documented as significant factors in integrated soil nutrient management for sustainable crop production as discussed throughout this review.

### Nitrogen-fixing biofertilizers

Nitrogen-fixing biofertilizers are currently the most common in the global market and their demand is anticipated to grow by a further 11.9% compounding annual growth rate (CAGR) to reach about USD 4.5 billion by 2026 up from the current USD 2.1 billion (Markets and Markets, 2020). Nitrogen-fixing biofertilizers comprise bacteria that carry out biological N_2_ fixation (BNF) and boost soil N supply to crops. The legume N_2_-fixing rhizobia have been researched for decades and shown to increase the quantity of fixed N in inoculated plants relative to un-inoculated ones (Koskey et al., 2017; Sanyal et al., 2020; Gedamu et al., 2021; Ketema and Tefera, 2022). Previous inputs of fixed N for red clover, alfalfa, soybean, pea, and cowpea were estimated to range from 23 to 335 kg ha^−1^ year^−1^ (Thies et al., 1995; Wani et al., 1995). The variabilities in terms of quantities of fixed N depend much on the type of legume-rhizobia symbiosis which is dictated by several factors like the legume cultivars and genotypes (Gunnabo et al., 2020; Riah et al., 2021), as well as the geographical distributions (Ramoneda et al., 2020).

According to Herridge et al. (2008), rhizobial inoculants can reduce the annual N fertilization costs by approximately USD 29 ha^−1^. This scenario demonstrates the importance of N_2_-fixing rhizobacteria as biofertilizers. Nevertheless, there is a need to perform field trials of new strains for suitability and adaptability before application as inoculants. Besides, N_2_-fixing biofertilizers are widely investigated for leguminous plants, and more efforts are required to demonstrate their potential in non-leguminous crops using asymbiotic diazotrophs like *Azospirillum, Azotobacter, Gluconaceotobacter*, and *Burkholderia*. Earlier studies by Melchiorre et al. (2011) and Hungria et al. (2006) both established that the yield of grains in Brazil, and Argentina, respectively, could reach close to 5 t ha^−1^ each season through rhizobia-mediated BNF. Similarly, annual N_2_ fixation rates of approximately 40 kg N ha^−1^ are documented in Australian soils (Unkovich and Baldock, 2008). Nevertheless, the contribution of asymbiotically fixed N in crop fields largely remains unestablished. More research is necessary, especially for crops like cereals, vegetables, and tubers, considering they contribute to the bulk of human food.

Some efficient N-fixing strains such as *Rhizobium* and *Azotobacter* spp. have successfully been formulated into commercial biofertilizers (Adeleke et al., 2019). However, the commercially available N biofertilizers mostly consist of *Rhizobium* and a few other bacteria such as *Azotobacter*, and *Azospirillum* species and are widely applicable to legume crops as presented in Section 4 (Vassilev et al., 2015; Adeleke et al., 2019). Nevertheless, inoculating crops and farms with such biofertilizers can meet the required N levels by plants and substantially reduce the application of artificial fertilizers (Aloo et al., 2022a).

### Phosphorus and potassium solubilizing biofertilizers

**Figure 3:**
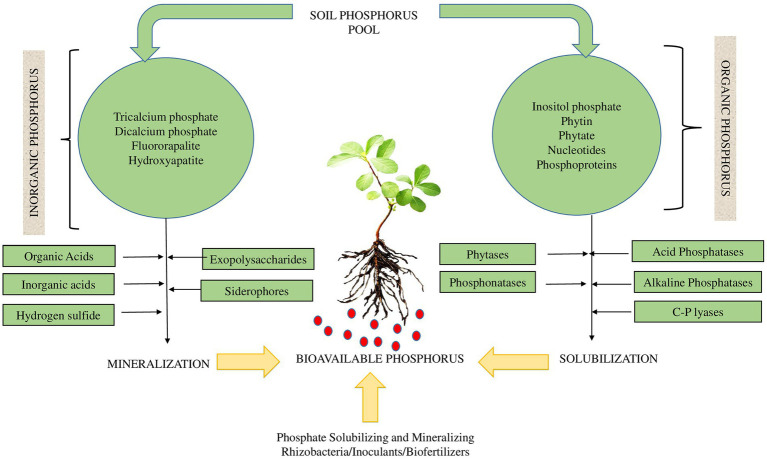
Phosphorus solubilization mechanisms depending on the types of available soil P.

Apart from N-fixation, biofertilizers can also solubilize plant nutrients in soil and facilitate their bioavailability and crop uptake (e.g., Ahmad et al., 2019; Abdelmoteleb and Gonzalez-Mendoza, 2020; El-Deen et al., 2020). Recent biofertilizer forecasts have favored the increased uptake of phosphatic biofertilizers owing to their ability to increase soil P availability and their biocontrol attributes for crop pests (Soumare et al., 2020). The solubilization of P is however dependent on the P forms in soil, whether organic or inorganic (Figure 3).

Since P deficiency is inherent in numerous agricultural soils, such organisms are largely proposed as potential P biofertilizers. Despite the growing literature, research concerning their application as biofertilizers is still limited and generally inconsistent. Since the economically-mineable P deposits are limited (Cordell et al., 2009; Vassilev et al., 2015), it is doubtless that phosphatic biofertilizers can significantly enhance crop yields, and that the use of P solubilizing biofertilizers (PSB) as bioinoculants can open up a new horizon for sustaining soil P levels and by large, sustainable crop production (Rizvi et al., 2021a).

Similarly, potassium solubilizing biofertilizers (KSB) are equally important in crop production since these are also often limiting in agricultural soils. The K-solubilizing capacity of PGPR from K-bearing rocks through acidification has widely been investigated, thus KSB have a significant role in enhancing crop growth and productivity, for instance, wheat (Laxita and Shruti, 2020), maize (Akintokun et al., 2019; Imran et al., 2020), tomatoes (Reyes-Castillo et al., 2019; Raji and Thangavelu, 2021), and many others. These reports show that these bacteria can significantly improve germination, uptake of nutrients, growth, and crop yields under both controlled and uncontrolled conditions. Although K solubilization may not entirely fulfill plant K requirements like chemical fertilizers, studies show that this novel approach may significantly improve K availability in croplands (Huda et al., 2007; Imran et al., 2020; Laxita and Shruti, 2020). Furthermore, the application of KSB to agricultural soils as biofertilizers can greatly cut the use of artificial fertilizers and are eco-friendly approaches to crop production. Native KSB are especially emerging as a viable technology for mitigating K deficits in agricultural soils. The diversity, solubilizing abilities, and mechanisms of KSB are extensively reviewed by Sattar et al. (2019) and Ahmad et al. (2016). Despite the burgeoning literature, little is still known about the efficacy of KSB and how they can stimulate plant growth in different climates. Meena et al. (2018) advance that KSB are valuable resources for mitigating K-deficiencies in agricultural farms but experimental results on their field efficacy are still grossly inadequate. More research is needed to enhance their usability. This, and related knowledge will undoubtedly help in comprehending their value as bioinoculants for practical field applications.

### Zinc solubilizing biofertilizers

Zinc solubilizing biofertilizers (ZSB) are equally important in crop production owing to worldwide Zn deficiency in soils. Such deficiency is prevalent in most arable lands caused by nutrient mining due to crop harvesting (Cakmak et al., 2017). Although chemical Zn fertilizers are often employed to augment these deficits at the recommended rates of approximately 5 kg ha^−1^ Zn, However, synthetic fertilizers are costly and do not readily get converted into plant-usable forms (Montalvo et al., 2016). Recent literature advances in rhizobacterial Zn solubilization (e.g., Joshi et al., 2013; Hussain et al., 2015; Kamran et al., 2017; Perumal et al., 2019) suggest that the field application of ZSB in the can increase Zn uptake by plants, and subsequently, improve their growth and yields. In an investigation by Naz et al. (2016), *Pseudomonas*, *Azotobacter*, *Azospirillum*, and *Rhizobium* species were shown to significantly enhance Zn uptake in wheat. Similarly, Sharma et al. (2012) studied 134 bacilli from the soybean (*G. max*) rhizosphere for Zn solubilization and established that the isolates greatly enhanced the concentration of Zn in the inoculated crops relative to the un-inoculated ones. Similarly, several ZSB like *Pantoea dispersa, P. fragi, P. agglomerans*, *Rhizobium* sp., and *E. cloacae* from the sugarcane and wheat rhizospheres were recently shown to improve the Zn contents and growth of potted wheat (Kamran et al., 2017). A more recent greenhouse trial by Dinesh et al. (2018) that evaluated several rhizospheric ZSB for their effects on soil and plant Zn contents revealed that the concentration in soil and plants was greater in treated plants than in non-treated ones. In India, Goteti et al. (2013) bacterized potted maize seeds with Zn-solubilizing *Pseudomonas* that significantly enhanced Zn uptake and concentration. Reports also exist for the Zn solubilizing abilities and increased Zn uptake following inoculation of wheat by Pseudomonads (Joshi et al., 2013), maize by *Bacillus* (Hussain et al., 2015), wheat and soybean by *B. aryabhattai* (Ramesh et al., 2014), and rice by several ZSB (Perumal et al., 2019).

### Iron sequestering biofertilizers

Some biofertilizers can sequester iron (Fe) through special mechanisms using metabolites called siderophores with a high affinity for Fe in low-Fe environments (Wang et al., 2022). After the formation of the Fe^3 + −^microbial siderophores complexes formed in the microbial membrane, the former is reduced to Fe^2+^ which is subsequently freed into the cell through an input mechanism. In this process, plants access and directly assimilate the Fe^2+^ from bacterial siderophores from the Fe-siderophore complexes or through ligand exchange reactions (Yehuda et al., 1996). Siderophore production is a typical example of Fe nutrition enhancement by rhizobacterial inoculants in biofertilizers and owing to its indisputable significance, should be given more attention (Aloo et al., 2019).

### Phytostimulators

**Figure 4:**
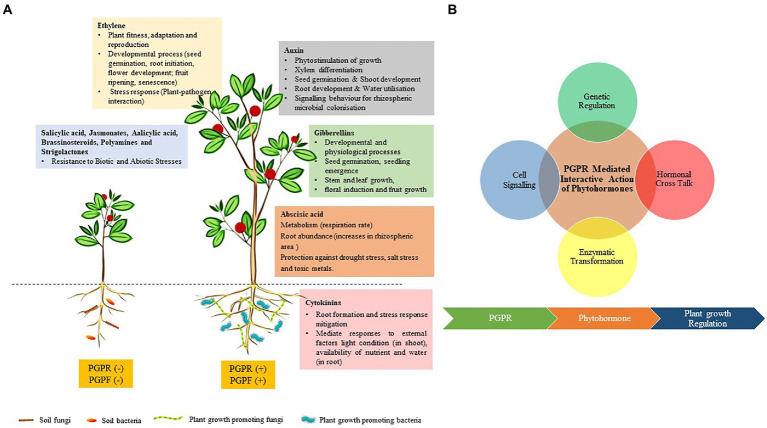
**(A)** Plant growth promotion through the production of different phytohormones and **(B)** PGPR-mediated plant growth promotion is governed through a complex network of cell signaling, genetic regulation, hormonal cross-talk, and enzymatic transformation. The PGPR generates multiple stimuli through the synthesis of phytohormones. These phytohormones interact through phosphorylation cascade or activating a secondary messenger which leads to the regulation of genes affecting hormone biosynthesis and developmental process in plants (Khan et al., 2020).

Still, other biofertilizers can promote plant growth through phytohormone production and plant growth stimulation in many ways (Figure 4). Such biofertilizers are largely known as phytostimulators owing to the various roles they play in stimulating the growth of crops through the production of phytohormones. The most common phytohormones are auxins, gibberellins, and cytokinin. Although very small amounts of phytohormones are produced by PGPR, they are still very crucial for plant metabolic processes, including those that modulate plant growth (e.g., Kalimuthu et al., 2019; Haerani et al., 2021) and plant tolerance to various abiotic stresses (Mahmoody and Noori, 2014; Santhi et al., 2021). Among the most potential PGPR that can function as biofertilizers due to phytohormone production are *Azospirillum* (e.g., Coniglio et al., 2019) and *Bacillus* spp. (Kang et al., 2019; Bandopadhyay, 2020), and many others. Owing to the importance of phytostimulation, such PGPR are viable candidates for PGP as biofertilizers, especially if they can also solubilize plant nutrients and/or fix N to improve plant nutrition.

## History of rhizobacterial biofertilizers

Whereas the application of microbial formulations is generally considered a modern and novel biotechnological agricultural approach in, crop inoculation with efficient PGPR for yield improvement is a century-old practice. The 1^st^ attempts at rhizobacterial formulation date back to the late 18^th^ century when a French scientist called Jean-Baptiste Boussingault (1801–1887) recognized that plant growth was proportional to N quantities. This observation was later linked to the reduction of N_2_ to ammonium and the 1st commercial biofertilizer Nitragin^®^ made from *Rhizobium* was produced (Nobbe and Hiltner, 1986). These were the first commercial formulations of PGPR that were patented and marketed over a century ago (Nobbe and Hiltner, 1986).

Due to the inconsistent performance of bioformulations relative to artificial fertilizers, the use of biofertilizers slowed down but picked up after subsequent decades of research that produced encouraging greenhouse results using *Pseudomonas* spp. (Kloepper et al., 1980). Large-scale field trials were performed using *Azotobacter* and *Bacillus* spp. on more than 35 million ha of land in the former Soviet Union in 1958 (Cooper, 1959), but the impact of bacterization was relatively unsatisfactory. Nevertheless, the commercialization of *Rhizobium* formulations continued in the 19th century (Fages, 1992), and extended globally thereafter (Deaker et al., 2004). A lot of biofertilizers have since been formulated and marketed worldwide attempts have also been made to formulate bacterial soil-fertilizing preparations for non-legume crops. The 1^st^ preparation “Alinit” based on *B. ellenbachensis* was introduced in Germany to promote cereal growth (Caron, 1897).

Rhizobacterial inoculations in parts of Southern Africa can be traced back to 1963 after successful soybean nodulation efficiency by native *Bradyrhizobium* and *Rhizobium* inoculants (Shurtleff and Aoyagi, 2018). Thereafter, a natural soybean nodulating variety called Nitrozam was formulated for use in Zambia and other African countries (Raimi et al., 2021). These concerted efforts massively increased soybean cultivation by 48% from 6,550 ha in 1984 to 22,780 ha in 1992. Additionally, about US$100,000 worth of Nitrozam was sold during this period. In South Africa, the biofertilizer market rapidly expanded in 1952, and to date, the country has one of the most established biofertilizer markets and regulations in the whole of Africa. The marketing and application of N_2_-fixing rhizobial biofertilizers in legume production have since been practiced for years. Globally, the total area of legumes under treatment with biofertilizers yearly was over 40 million ha by the year 2000 (Phillips, 2004), half of which was used for soybean production (Catroux et al., 2001). There are more success stories of legume inoculants in different parts of the world (El-Wakeil and El-Sebai, 2009; Ngakou et al., 2009; Gomare et al., 2013). The production and marketing of rhizobial inoculants for legume production have thus been practiced for decades, somewhat decreasing the need for chemical fertilizers in several countries around the world. The development of new biofertilizer bioformulations continues to expand, and the future of the technology seems bright.

## The current state of rhizobacterial biofertilizers

There is a burgeoning literature on the current application of microbial products as biofertilizers and agricultural inputs. Nearly 170 establishments in 24 countries commercialize biofertilizers and possess factories that produce, and market microbe-based fertilizers at both small and large scales (Bharti et al., 2017). The marketing of rhizobial inoculants has particularly been practiced for several decades now to partially eliminate the application of artificial fertilizers (Paudyal and Gupta, 2018). However, the full potential of many potential biofertilizers is largely untapped. Likewise, biofertilizer commercialization remains low globally, albeit steadily increasing.

In developed countries where artificial agricultural inputs are fairly cheap, the use of PGPR is less prioritized but is albeit growing. In 2013, the highest demand for biofertilizers was highest in North America and projections were that the entire Asia-Pacific biofertilizer market would show the maximum growth from 2014 to 2019 and lead in biofertilizer consumption worldwide (Markets and Markets, 2014). The consumption of biofertilizers is reportedly growing in countries such as Canada, Argentina, China, India, Europe, and the United States of America (USA) due to tax exemptions, and input subsidies, among other incentives (Markets and Markets, 2019). Such approaches have generally served to expand the global biofertilizer market, but more efforts are still required.

The advancement of research around the globe on the diversity, functions, and potentials of native rhizobacteria has stimulated the selection and isolation of efficient PGPR, and several biofertilizer formulations are already produced and commercialized for use in different countries across the globe (Table 2). The most progressive and dominant biofertilizer market in the world is Europe, where biofertilizer demand has grown at a CAGR of 12.3% from approximately US$2566 million in 2012 to US$4582 million in 2017 (Chandrasekhar, 2014). The global biofertilizer market was worth US$1.06 million in 2016 and was estimated to hit US$2 billion in 2019 and over US$3.8 billion in 2026, at a CAGR of 11.2% (Markets and Markets, 2020). The global increase in demand for biofertilizers has greatly been influenced by the growing demand for organic food products.

**Table 2:** Examples of commercial biofertilizer products in some countries around the world.

Country	Product	Organisms	Manufacturer	Crop	References
Argentina	Liquid PSA	*P. aurantiaca*	Laboratorios BioAgro S.A.	Wheat	Celador-Lera et al. (2018)
	Zadspirillum	*Azospirillum brasilense*	Semillera Guasch SRL	Maize	Celador-Lera et al. (2018)
	Rhizo Liq	*Bradyrhizobium* sp.*, Mesorhizobium ciceri, Rhizobium* spp.	Rhizobacter	Chickpea, Soybean, Common bean, green gram, Groundnut	Adeleke et al. (2019)
Australia	Bio-N	*Azotobacter* spp.	Nutri-Tech solution	Not stated	Adeleke et al. (2019)
	Myco-Tea	*Azotobacter chroococcum, B. polymyxa*	Nutri-Tech solution	Tea	Adeleke et al. (2019)
	Twin N	*Azorhizobium* sp.*, Azoarcus* sp.*, Azospirillum* sp.	Mapleton Int. Ltd	Not stated	Adeleke et al. (2019)
Brazil	Bioativo	PGPR consortia	Embrafros Ltda	Beans, maize, sugarcane, rice, cereals	Odoh et al. (2019)
Canada	Rhizocell GC Nodulator	*B. amyloliquefaciens IT 45, B. japonicum*	Lallen and plant care BASF Inc.	Beans, maize, carrot, rice, cotton	Odoh et al. (2019)
	Vault HP	*Bradyrhizobium* sp.	BASF	Not stated	Adeleke et al. (2019)
China	CBF	*Bacillus mucilaginosus, B. subtilis*	China Bio-Fertilizer AG	Various cereals	Celador-Lera et al. (2018)
Colombia	Fe Sol B	*Not mentioned*	Agri Life Bio Solutions	Not stated	Mishra and Arora (2016)
Germany	FZB 24 fl, BactofilA 10	*B. amyloliquefaciens, B. megaterium, P. fluorescens*	AbiTEP GmbH	Vegetables, cereals	Odoh et al. (2019)
Hungary	BactoFil A10	*A. brasilense, Azotobacter vinelandii, B. megaterium*	AGRObio	Maize	Mustafa et al. (2019)
India	Ajay *Azospirillum*	*Azospirillum*	Ajay Biotech	Cereals	Celador-Lera et al. (2018)
	Greenmax AgroTech Life Biomix, Biodinc, G max PGPR	*Azotobacter, P. fluorescens*	Biomax	Various crops	Odoh et al. (2019)
	Fe Sol B	*Not mentioned*	Agri Life Bio Solutions	Not mentioned	Mishra and Arora (2016)
	Symbion van plus	*B.megaterium*	T. Stanes and Co. Ltd	Not mentioned	Celador-Lera et al. (2018)
Kenya	Biofix	Rhizobia	MEA Fertilizer Ltd	Not mentioned	Adeleke et al. (2019)
	Kefrifix	Not mentioned	KFRI	Not mentioned	Raimi et al. (2021)
Nigeria	Nodumax	*Bradyrhizobia*	IITA	Not mentioned	Tairo and Ndakidemi (2014), Adeleke et al. (2019)
Russia	*Azobacterium*	*Azobacterium brasilense*	JSC Industrial Innovations	Wheat, barley, maize,	Celador-Lera et al. (2018)
South Africa	Organico	*Rhizobium, Enterobacter* spp., *Bacillus* spp., *Stenotrophomonas, Pseudomonas*	Amka Products (Pty) Ltd	Not mentioned	Adeleke et al. (2019)
	Azo-N, Azo-N-Plus	*A.brasiliense, A. lipoferum*	Biocontrol Products Ltd	Not mentiomne	Raimi (2018)
	Lifeforce, Firstbase, Biostart, Landbac, Composter, Waterbac	*Bacillus* spp.,	Microbial solution (Psty) Ltd	Not stated	Mohammadi and Sohrabi (2012)
	Histick	*B. japonicum*	BASF	Not stated	Tairo and Ndakidemi (2014)
	N-Soy	*B.japoniucm*	Biocontrol Products Ltd	Not stated	Tairo and Ndakidemi (2014)
	Soilfix	*Brevibacillus laterosporus, Paenibacillus chitinolyticus*	Biocontrol Products Ltd	Not stated	Grady et al. (2016)
	Organico	*Bacillus* sp.	Amka Products	Not stated	Raimi (2018)
	Bac-up	*B. subtilis*	Biocontrol Products Ltd	Not stated	Adeleke et al. (2019)
Spain	InomixR	*B. polymyxa, B. subtilis*	Lab (Labiotech)	Cereals	Odoh et al. (2019)
	Vita Soil	*PGPR consortia*	Symborg	Not stated	Sekar et al. (2016)
Thailand	BioPlant	*Streptomyces, Nitrobacter, Clostridium, Bacillus, Aerobacter, Achromobacter, Nitrosomonas*	Artemis & Angelio Co. Ltd.	Not stated	Adeleke et al. (2019)
United Kingdom	Ammnite A 100	*Azotobacter, Bacillus, Rhizobium, Pseudomonas*	Cleveland biotech	Cucumber, tomato, pepper	Odoh et al. (2019)
	Legume Fix	*Rhizobium* sp.*, B. japonicum.*	Legume Technology	Common bean, Soybean	Adeleke et al. (2019)
	Twin N	*Azorhizobium* sp.*, Azoarcus* sp.*, Azospirillum* sp.	Mapleton Int. Ltd	Not mentioned	Adeleke et al. (2019)
Uruguay	Nitrasec	*Rhizobium* sp.	Lage y Cia	Not mentioned	Adeleke et al. (2019)
United States	Inogro	*30 bacterial species*	Flozyme Corporation	Rice	Celador-Lera et al. (2018)
	Vault NP	*B. japonicum*	Becker Underwood	Not mentioned	Adeleke et al. (2019)
	Chickpea Nodulator	*Mesorhizobium cicero*	Becker Underwood	Chickpea	Adeleke et al. (2019)
	Cowpea Inoculant	*Rhizobia*	Becker Underwood	Cowpea	Adeleke et al. (2019)
	PHC Biopak	*B. subtilis, B. azotofixans, B. megaterium, B. licheniformis, B. thuringiensis, B. polymyxa,*	Plant Health Care Inc.	Not mentioned	Adeleke et al. (2019)
	Complete Plus	*Bacillus strains*	Plant Health Care	Various crops	Mustafa et al. (2019)
	Quickroots	*B. amyloliquefaciens*	Monsanto	Wheat, common bean	Celador-Lera et al. (2018)

Although there exist many reports on the formulation and/or commercialization and application of rhizobacterial biofertilizers in several parts of the world, only a few reports indicate their application and commercialization in Africa. The PGPR inoculant technology has little or no impact on crop productivity in developing countries since it is either not practiced or the poor quality of available inoculants (Bashan, 1998). According to Aloo et al. (2021), the potential benefits of biofertilizers have largely been untapped in Africa due to inadequate regulatory frameworks and several other challenges. Most biofertilizers are commercialized for use in Asia, Europe, and the USA but in Africa, most commercialization and application occur only in South Africa. In East Africa, the production and use of biofertilizers are pronounced in Kenya which is the manufacturer of Biofix which can effectively inoculate 15 kg of common bean seeds per ha at approximately US$1.25 in comparison to 90 kg of artificial N fertilizer required for the same number of seeds per ha at US$12.50 (Raimi et al., 2021). However, Biofix and other biofertilizers are still largely underutilized in Kenya, probably due to a lack of awareness and other technology adoption challenges. Current and future initiatives are anticipated to improve the uptake of biofertilizers in Africa (Raimi et al., 2021). However, more efforts are needed to boost the consumption of these microbial products and promote the sustainability of global food production systems (Aloo et al., 2021).

The worldwide market for biofertilizers is presently largely dominated by legume and N_2_-fixing inoculants (Vassilev et al., 2015). While rhizobial inoculants currently dominate the global biofertilizer market, PSB, and other bioinoculants occupy less than 30% altogether (Transparency Market Research, 2022). Nevertheless, P-, K-, and Zn-based biofertilizers are now developing into significant bioinoculants to address soil nutrient deficiencies, and KSM are already commonly used as inoculants in some countries with K-deficient croplands (Teotia et al., 2016). India is reportedly the 4th largest consumer of K bioinoculants globally while countries like Brazil, the USA, and China come first in the overall consumption of these microbial products (Matich, 2016).

Unlike rhizobial biofertilizers, PSB like *Pseudomonas, Bacillus*, and diazotrophs like *Azospirillum* have neither been used as much nor at a large scale (Lesueur et al., 2016). Most of the commercially-available non-rhizobial PGPR inoculants consist of *Azospirillum* as free-living N_2_ fixers or Bacilli as PSB (Herrmann and Lesueur, 2013). The application of non-rhizobial biofertilizers has a less significant impact on global food production probably because of several bottlenecks that exist in biofertilizer uptake and use relative to well-documented PGP functions. Yet, PGPR like PSB are essential candidates for improving legume P nutrition for efficient nodulation (Zaidi et al., 2017). The global biofertilizers market for crop production is projected to grow from US$2.02 billion in 2022 to US$4.47 billion by 2029, at a CAGR of 12.04% from 2022 to 2029 (Fortune Business Insights, 2022). Still, there is a need for more efforts for adequate market infiltration and application.

## The future of biofertilizers

The incorporation of biofertilizers as fundamental components of agricultural practices is quickly gathering momentum globally. These microbial products are already in use in some countries and are expected to become more popular in the future. The global and future expansion of the biofertilizer market will largely be driven by the need to increase food production sustainably. With an ever-increasing demand for organic food products, growing awareness of sustainable agricultural practices, and promotion of cleaner production methods for reducing soil contamination, land degradation, and water pollution the market growth for biofertilizers will continue to grow over the coming years (Abhilash et al., 2016a; Anand et al., 2022; Vaishnav et al., 2022).

Forecasts are that the present global market for biofertilizers which was approximated at US$396 million in 2018 will grow at a CAGR of 10.9% to escalate to US$4448.97 million by 2028 (Vintage Market Research, 2022). Further indications are that the global biofertilizer market which was valued at close to US$ 3.0 billion in 2020 will grow at a CAGR of 12.2% and reach about US$5 billion by 2031 (Transparency Market Research, 2022). The number of investigations targeting the isolation, identification, and evaluation of the capacity of PGPR with the potential of being transformed into inoculants for a variety of crops is equally expanding (Vatsyayan and Ghosh, 2013; Datta et al., 2015; Tan et al., 2015; Koskey et al., 2017; Anand et al., 2022; Aloo et al., 2022b). It is, therefore, realistic to expect that widespread biofertilizer usage will soon offer countless approaches to the progression of sustainable crop production systems.

For the extensive utilization of biofertilizers, proper regulatory and legal frameworks will be required in place of the existing ones that are currently stringent and hinder their proper utilization. Fortunately, regulatory authorities are increasingly encouraging the implementation of alternative crop fertilization mechanisms to promote the development of sustainable agricultural technologies. Recognizing the need for a specific legislative framework for biofertilizers in Europe, the European Commission has proposed to amend existing regulations (European Parliament and Council of the European Union, 2016). Such initiatives will eventually relax the stringent regulatory frameworks and enable the widespread adoption of these microbial resources.

**Figure 5:**
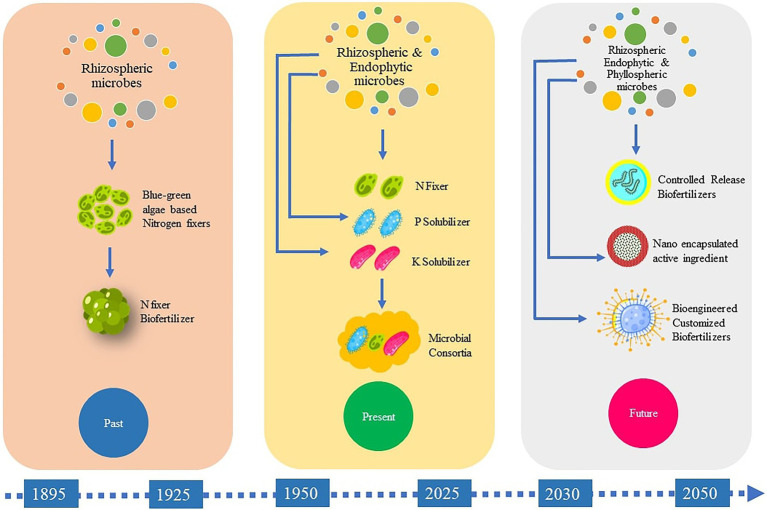
Schematic representation of the past present and future of biofertilizer development.

While a number of the existing biofertilizers are mainly composed of natural rhizobacterial strains chosen for their PGP qualities, the development of genetically-modified inoculants that are likely to be more efficient at plant growth stimulation is required. Still, the biggest hurdle will be for scientists to convince society and regulatory authorities worldwide that such genetically-engineered organisms are harmless. Our current ability to manipulate and exploit the plant microbiome *in situ* similarly remains limited, and more investigations are required to facilitate their large-scale application and commercialization. The inoculant industry is faced with various challenges in making formulations with prolonged shelf lives. Progress into developing formulations with improved shelf lives, broad spectra of action, and reliable field performance will therefore hasten the commercialization of this technology (Nakkreen et al., 2005). In this regard, new approaches should be evaluated to develop formulations with longer shelf lives. Micro-encapsulation is one viable approach but most experiments in this regard have been restricted to laboratories and the standardization of this technology for industrial and field applications should be pursued. The future of biofertilizer technology depends a lot on developing efficient PGP strains. This is quite challenging but continued research in this area will eventually pave the way for this (Figure 5).

Investigation on N-fixing and PSB is developing fairly well, unlike for K solubilizers despite K being among the important macronutrients for plant development. More research in this regard will promote their application and utilization as bioinoculants in the future. Future research should additionally focus on the development of inoculants that can tolerate unfavorable environmental conditions for applicability in stressful environments, especially for inoculants that have been shown to relieve plants of metal stress (Rizvi et al., 2019, 2020, 2022b), salinity stress (Shahid et al., 2021), and cold stress (Rizvi et al., 2021b). More research is needed on the practical aspects of large-scale formulation and production to develop stable, effective, and state-of-the-art bioformulations. Microbial consortia offer multiple PGP traits for producing novel biofertilizer formulations as substitutes for artificial inputs (Singh et al., 2020; Cakmaksi et al., 2021; Vaishnav and Singh, 2021).

The interactions among plants and biofertilizer inoculants will require further studies and new approaches. Future research should additionally include careful selection of rhizosphere microbiota, and their *in-situ* testing for use as plant inoculants. It is expected that the identification of effective microbiomes in different soil types and climates will be extremely helpful in this regard. To improve this strategy, establishing a global database of effective plant microbiomes will be an important milestone toward successful translational research. A lot of obstacles remain to be overcome before this can fully be realized. For instance, several formulations based on such microbes have been developed for applications to different crops worldwide. However, inoculation results are often inconsistent and dependent on the prevailing local soil and plant-related properties, altogether necessitating the optimization of each system.

The application of biotechnology and the improvement of biofertilizer regulations will facilitate in designing of more effective and reliable rhizobial bioformulations as biofertilizers. To design suitable rhizobial formulations, we must use modern technologies to increase our understanding of plant-microbe interactions (Jain et al., 2021). For example, multi-omics approaches can greatly help us to comprehend complex plant-microbial symbioses to design suitable bioformulations for particular soils and crops (Kaul et al., 2016; López-Mondéjar et al., 2017; Ding et al., 2021; Yamazaki et al., 2021). These novel approaches will with time enhance the complete characterization of PGPR and their influence on plant nutrient acquisition and other PGP traits to facilitate their application. Thus, these should be prioritized for research.

Finally, it will be important to identify the challenges in the production and application of biofertilizers and strategies to address such problems. For example, the field efficiency of biofertilizers is dependent on crop species, soil complexity, and climatic conditions. Research on suitable biofertilizers should in the future be handled by agronomists that understand the nexus between crops, climatic conditions, and nutrients in various parts of the world. Besides, genomic engineering can be necessary for manipulating indigenous PGPR with suitable genes for enhanced expression of biofertilization functions for field applications. Additionally, particular additives could improve product stability, shelf life, and field efficiency.

## Conclusion

The greatest global challenge in the 21st century is to invent and implement sustainable agricultural practices. This can only be achieved if we accommodate changing and advanced technologies such as the use of efficient rhizobacterial biofertilizers. The present discussion is useful for the development of sustainable agricultural systems. The use of these bio-resources though has been practiced in several parts of the globe is still low but the results are encouraging and there is room for development to boost their efficiency. With time, the practice will certainly grow and projections are that biofertilizers will have massive market potential soon. Researchers, agricultural institutions, and universities can fast-track biofertilizer development and promote their usage and adaptation for sustainable agricultural practices. If issues linked to regulation, policy development, and social acceptability of biofertilizer products can simultaneously be addressed, these bio-based tools can potentially and significantly contribute to sustainable agricultural productivity.

## Author contributions

All authors listed have made a substantial, direct, and intellectual contribution to the work and approved it for publication.

## Conflict of interest

The authors declare that the research was conducted in the absence of any commercial or financial relationships that could be construed as a potential conflict of interest.

## Publisher’s note

All claims expressed in this article are solely those of the authors and do not necessarily represent those of their affiliated organizations, or those of the publisher, the editors and the reviewers. Any product that may be evaluated in this article, or claim that may be made by its manufacturer, is not guaranteed or endorsed by the publisher.
